# Quercetin Directly Targets JAK2 and PKCδ and Prevents UV-Induced Photoaging in Human Skin

**DOI:** 10.3390/ijms20215262

**Published:** 2019-10-23

**Authors:** Eun Ju Shin, Ji Su Lee, Seungpyo Hong, Tae-Gyu Lim, Sanguine Byun

**Affiliations:** 1 Korea Food Research Institute, Wanju-gun, Jeollabuk-do 55365, Korea; Shin.Eun-ju@kfri.re.kr (E.J.S.);; 2 Division of Bioengineering, Incheon National University, Incheon 22012, Korea; 201821148@inu.ac.kr

**Keywords:** quercetin, skin aging, PKC-delta, JAK2

## Abstract

Quercetin is a naturally occurring polyphenol present in various fruits and vegetables. The bioactive properties of quercetin include anti-oxidative, anti-cancer, anti-inflammatory, and anti-diabetic effects. However, the effect of quercetin on skin aging and the direct molecular targets responsible have remained largely unknown. Herein, we investigated the protective effect of quercetin against UV-mediated skin aging and the molecular mechanisms responsible. Treatment with quercetin suppressed UV-induced matrix metalloproteinase-1 (MMP-1) and cyclooxygenase-2 (COX-2) expression and prevented UV-mediated collagen degradation in human skin tissues. Quercetin exerted potent inhibitory effects towards UV-induced activator protein-1 (AP-1) and nuclear factor-kappa B (NF-κB) activity. Further examination of the upstream signaling pathways revealed that quercetin can attenuate UV-mediated phosphorylation of extracellular signal-regulated kinase (ERK), c-Jun N terminal kinases (JNK), protein kinase B (Akt), and signal transducer and activator of transcription 3 (STAT3). Kinase assays using purified protein demonstrated that quercetin can directly inhibit protein kinase C delta (PKCδ) and Janus kinase 2 (JAK2) kinase activity. Quercetin was observed to bind to PKCδ and JAK2 in pull-down assays. These findings suggest that quercetin can directly target PKCδ and JAK2 in the skin to elicit protective effects against UV-mediated skin aging and inflammation. Our results highlight the potential use of quercetin as a natural agent for anti-skin aging applications.

## 1. Introduction

The process of skin aging involves both endogenous and exogenous aging and is generally associated with deep wrinkles, pigmentation, sagging, and laxity [1,2]. Chronic or excessive exposure to UV radiation can promote epidermal inflammation, wrinkle formation, and tumorigenesis [3]. UV exposure to the skin causes the induction of matrix metalloproteinases (MMPs), particularly MMP-1, which mediates the degradation of collagen leading to skin aging [3,4]. Cyclooxygenase 2 (COX-2) is an inducible enzyme functioning as a pro-inflammatory mediator which has been reported to participate in skin photoaging and skin cancer development [5]. UV-driven upregulation of enzymes such as MMP-1 and COX-2 has been known as a major factor promoting skin aging and thus has been recognized as a therapeutic target.

UV radiation activates a variety of signaling molecules involved in the regulation of skin aging [6]. Exposure to UV induces the activation of Protein kinase C (PKC) family members including PKC-delta (PKCδ), which in turn interact with the mitogen-activated protein kinase (MAPK) pathways [7,8]. Activation of MAPKs—including extracellular signal-regulated kinase (ERKs), c-Jun N terminal kinases (JNKs), and p38—is closely associated with the UV-mediated skin aging process [6,9]. Specifically, MAPKs can stimulate transcription factors such as nuclear factor-kappa B (NF-κB) and activator protein-1 (AP-1), which promote the degradation of the extra cellular matrices via upregulation of MMPs and COX-2 expression and repress collagen production in the skin [10,11]. In addition, Janus kinase 2/signal transducer and activator of transcription 3 (JAK2/STAT-3) signaling pathway plays a critical role in skin inflammation [12,13]. These studies provide evidence that PKCδ and JAK2 act as key intermediates in UV-mediated signaling, and the potential to be therapeutic targets in preventing skin-aging.

Quercetin (3,3′,4′,5,7-pentahydroxyflavone), a well-known member of the flavonoid family, has been reported to elicit a variety of beneficial effects, including anti-oxidant, anti-inflammatory, and anti-carcinogenic activities [14,15]. However, the anti-skin aging efficacy of quercetin and its direct molecular targets are not fully understood. In the present study, we sought to investigate the mechanisms of action responsible for the effects of quercetin against UV-induced skin aging in skin cells and human skin tissue.

## 2. Results

### 2.1. Quercetin Prevents UV-Induced Skin Aging in Human Skin Tissue

**Figure 1:**
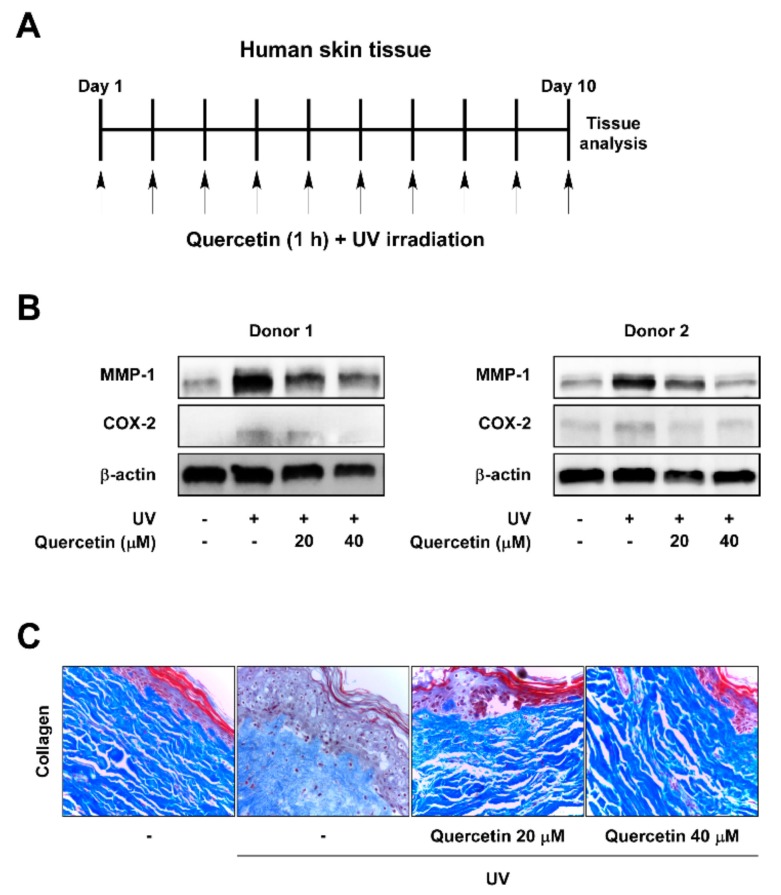
Quercetin suppresses MMP-1 and COX-2 expression and blocks collagen degradation in UV-irradiated human skin tissue. (**A**) Human skin tissue samples were pre-treated with quercetin at the indicated concentrations for 1 h before being exposed to UV (26 kJ/cm^2^) over 10 days. Skin tissues were subjected to UV at the same time each day. (**B**) Protein expressions of MMP-1, COX-2, and β-actin were determined in human tissue lysate by immunoblot analysis. (**C**) Collagen fibers were detected using Masson’s trichrome staining.

To investigate the protective effects of quercetin against UV-induced skin aging, human skin tissues were treated with quercetin and irradiated with UV once a day for 10 successive days in ex vivo conditions (Figure 1A). As MMP-1 functions as a key enzyme in skin wrinkle formation [3], we first examined the inhibitory effect of quercetin against UV-induced MMP-1 levels. UV irradiation increased MMP-1 expressions, which was suppressed by treatment with quercetin in human skin tissues (Figure 1B). Because UV also mediates skin aging through promoting inflammatory responses, we evaluated the effects of quercetin on COX-2 expression, a major inducer of inflammation [5]. Quercetin attenuated UV-induced COX-2 expression in human skin tissues (Figure 1B). Analysis of collagen content demonstrated that quercetin can prevent UV-induced collagen degradation in human skin tissues (Figure 1C). These results indicate that quercetin has anti-wrinkle and anti-inflammatory effects in human skin tissue.

### 2.2. Quercetin Inhibits UV-Induced AP-1 and NF-κB Activation

**Figure 2:**
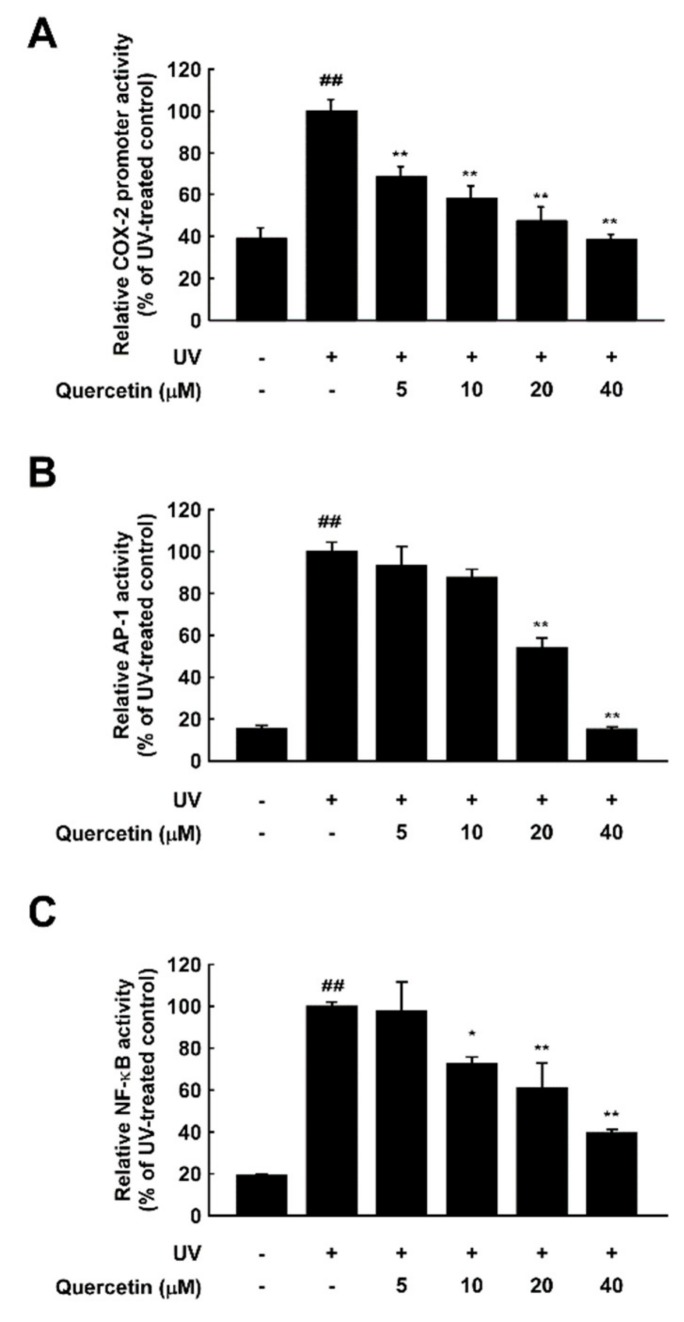
Inhibitory effect of quercetin against UV-induced COX-2 promoter activity and AP-1 and NF-κB activation. Cells stably expressing COX-2 promoter reporter or AP-1 or NF-κB reporter plasmids were used. Quercetin was treated at the indicated concentrations for 1 h prior to UV irradiation. Luciferase activity was measured for (**A**) COX-2, (**B**) AP-1, and (**C**) NF-κB. ## indicate significant (*p* < 0.001) induction by UV compared to the un-treated control. *,** indicate significant (*p* < 0.05, and *p* < 0.001, respectively) inhibition of activity by quercetin compared to the UV-only group.

To determine whether the inhibitory effects of quercetin on UV-induced skin aging are mediated by transcriptional regulation, we examined UV-induced *cox-2* promoter activity with a luciferase reporter assay in JB6 P+ epidermal cells. Quercetin significantly reduced UV-induced *cox-2* promoter activity in a dose-dependent manner (Figure 2A). Previous studies have shown that UV irradiation activates several transcription factors including AP-1 and NF-κB, which subsequently induces MMP-1 and COX-2 expression [10,11]. We next investigated whether quercetin affects AP-1 or NF-κB activation using cells stably transfected with a NF-κB or AP-1 luciferase reporter plasmid. Quercetin significantly inhibited UV-induced activation of both targets (Figure 2B,C). These results demonstrate that quercetin inhibits UV-induced skin aging by suppressing the AP-1 and NF-κB.

### 2.3. Quercetin Suppresses UV-Induced Phosphorylations of ERK, JNK, Akt, and STAT3

**Figure 3:**
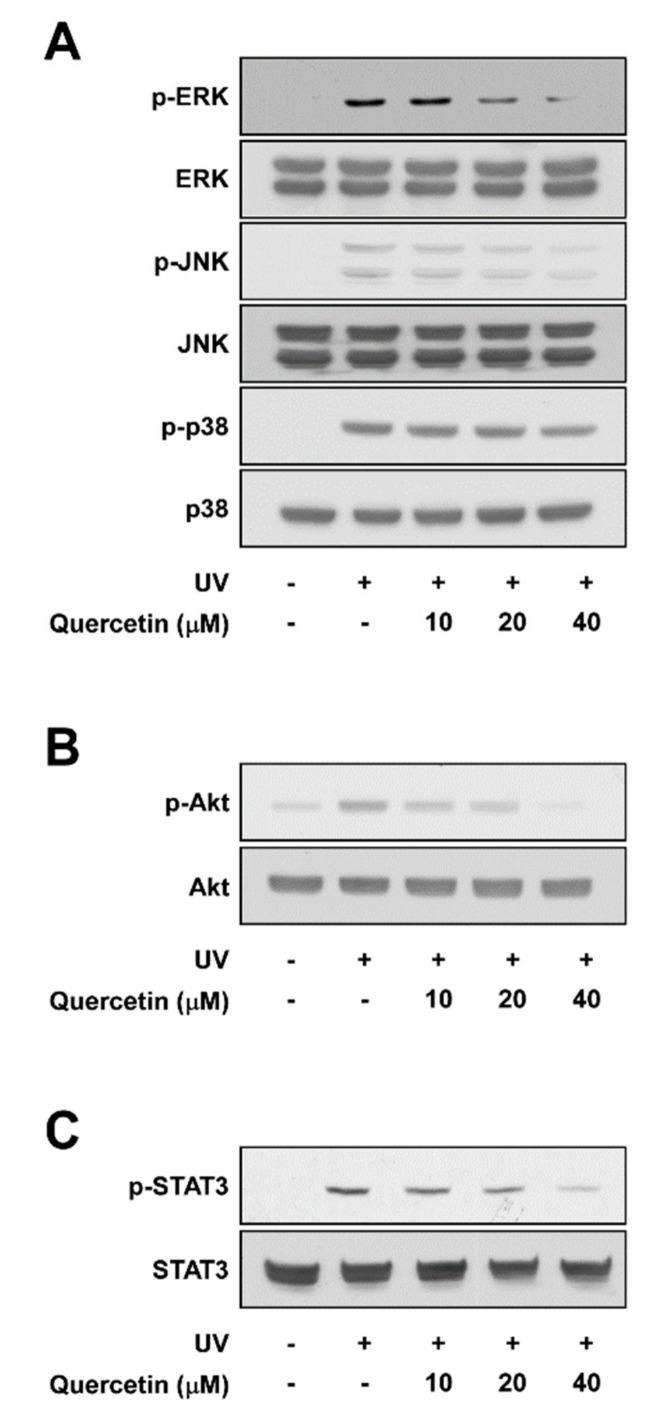
Effect of quercetin on UV-induced signaling pathways. Cells were pre-treated with quercetin at the indicated concentrations for 1 h, before exposure to UV irradiation. Protein levels of phosphorylated and total (**A**) ERK, JNK, p38; (**B**) Akt; and (**C**) STAT3 were assessed in cell lysates by immonoblot. Immunoblots are representative images of three independent experiments.

The MAPK family (ERK, JNK, and p38), Akt, and STAT3 signaling pathway are well-known upstream regulators of AP-1 and NF-κB [16,17]. Pre-treatment with quercetin attenuated UV-induced phosphorylation of ERK and JNK in a dose-dependent manner, while displaying no effect towards p38 phosphorylation (Figure 3A). Quercetin also reduced UV-induced phosphorylations of Akt and STAT3 (Figure 3B,C). These findings suggest that quercetin may attenuate UV-stimulated COX-2 and MMP-1 expression by suppressing ERK, JNK, Akt, and STAT3 signaling.

### 2.4. Expression of Dominant Negative PKCδ Suppresses UV-Induced MAPK and Akt Activation

**Figure 4:**
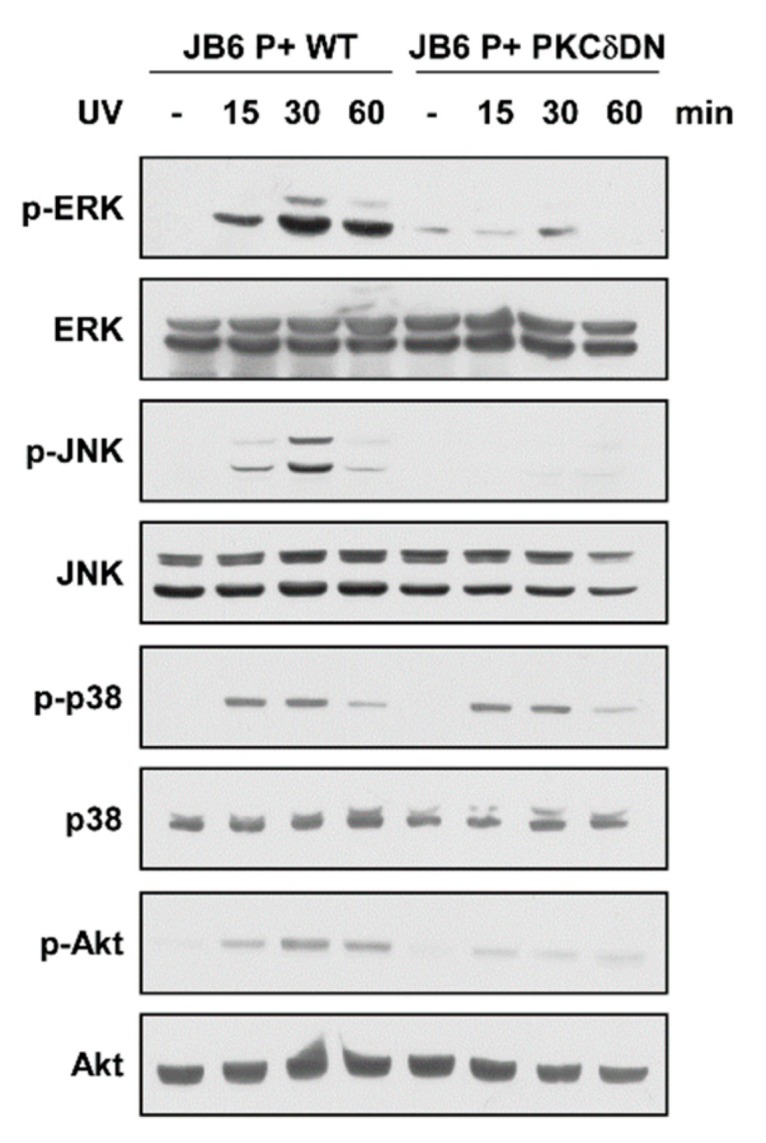
Effect of PKCδ on UV-induced signaling pathways. Cells were transfected with either a mock vector or a dominant-negative mutant (DN) of the PKCδ. Following incubation, proteins were extracted with cell lysis buffer. Protein levels of phosphorylated and total ERK, JNK, p38, and Akt were examined by immunoblot. Immunoblots are representative images of three independent experiments.

Previous reports suggest that the PKC family, particularly PKCδ, may act as upstream regulators of MAPK and Akt [18]. To elucidate the role of PKCδ in regulating UV-induced signaling pathways, we examined MAPK (ERK, JNK, and p38) and Akt phosphorylations after UV irradiation in cells expressing dominant negative PKCδ (PKCδ-DN). UV irradiation increased phosphorylation levels of ERK, JNK, p38, and Akt with peak induction at 30 min (Figure 4 and Figure S1A). In contrast, inhibition of PKCδ activity by expressing PKCδ-DN suppressed UV-stimulated phosphorylations of ERK JNK, and Akt, but not the phosphorylation of p38 (Figure 4). Cells expressing PKCδ-DN also showed reduced MMP-13 and COX-2 expression levels (Figure S1B). These results show that PKCδ functions as an upstream regulator of ERK, JNK, and Akt in UV signaling pathway and reflects the changes seen after quercetin treatment.

### 2.5. Quercetin Directly Binds to and Attenuates the Activity of PKCδ and JAK2 Kinase

**Figure 5:**
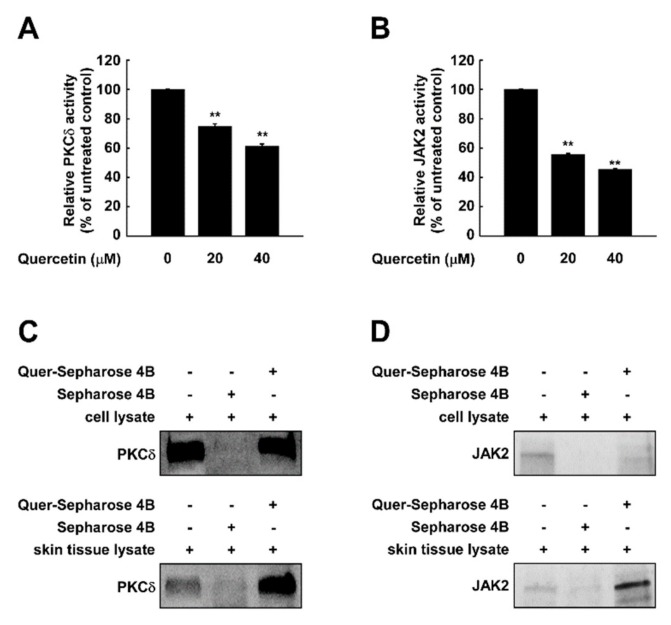
Quercetin binds to and inhibits PKCδ and JAK2 kinase. Kinase assays was performed with (**A**) PKCδ or (**B**) JAK2 protein. Pull-down assays were performed to detect the binding of quercetin with PKCδ or JAK2. Binding between PKCδ-and quercetin or between JAK2- and quercetin were confirmed in JB6 P+ cells and human skin tissues by immunoblotting using antibodies against (**C**) PKCδ or (**D**) JAK2. Lane 1: whole lysates isolated from cell or tissue; lane 2: lysates of cell or tissue samples were precipitated with Sepharose 4B-alone; lane 3: whole cell lysates from cell or tissue were precipitated with quercetin-Sepharose 4B beads (Quer-Sepharose 4B). ** indicate significant (*p* < 0.001) inhibition of activity by quercetin compared to un-treated control.

As suppressing PKCδ displayed similar signaling patterns to that of quercetin treatment, we questioned whether quercetin affects PKCδ activity. To examine the effect of quercetin on PKCδ kinase activity, we examined the in vitro kinase activity using purified PKCδ. Quercetin inhibited PKCδ activity in a dose-dependent manner (Figure 5A). In addition, as STAT3 is a direct substrate of JAK2, we also examined if quercetin targets JAK2. Treatment with quercetin reduced the activity of JAK2 kinase (Figure 5B). A pull-down assay using quercetin-conjugated sepharose 4B beads showed that quercetin directly interacts with PKCδ isolated from both skin cells and tissue extracts (Figure 5C). Also, quercetin-sepharose 4B beads, but not sepharose 4B beads alone, bound JAK2 from skin cells and human skin tissue extracts (Figure 5D). These results indicate that quercetin can bind to JAK2 and PKCδ in the skin and inhibit their kinase activities.

### 2.6. Docking Model of Quercetin with PKCδ and JAK2

**Figure 6:**
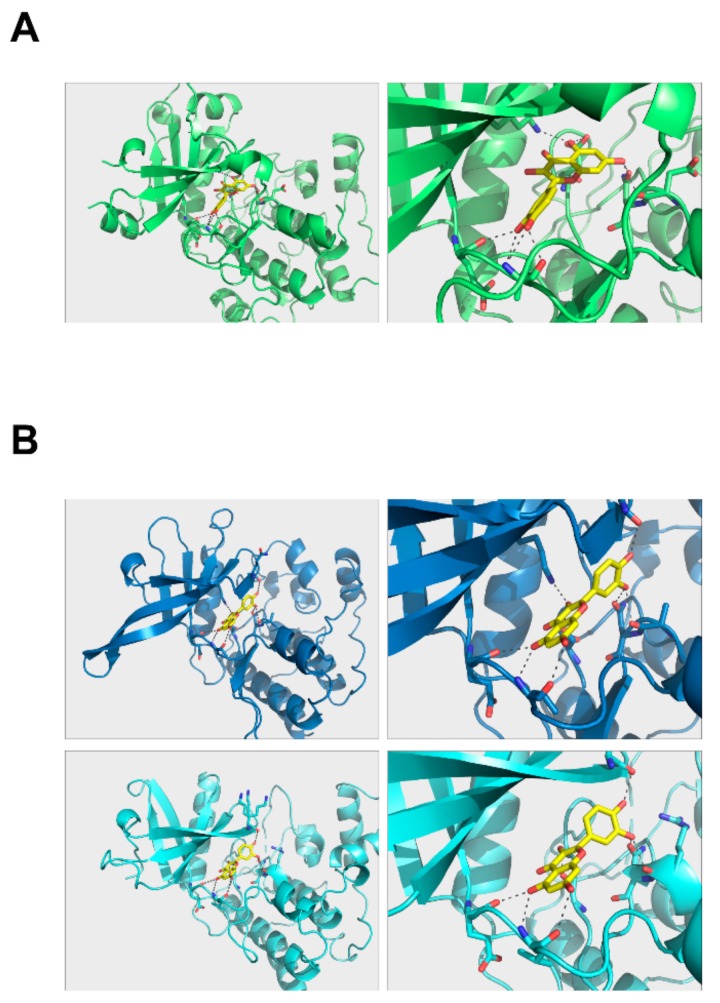
Quercetin complex structure models. (**A**) PKCδ and (**B**) JAK2 protein kinase 1 (upper panel), JAK2 protein kinase 2 (lower panel). Autodock scores for PKCδ, JAK2-PK1, and JAK2-PK2 = −8.9, −8.9, and −9.3 kcal/mol, respectively.

To further investigate how quercetin binds to PKCδ and JAK2, we modeled the structure of quercetin in complex with PKCδ and JAK2. Quercetin can be placed in the hydrophobic region of their kinase domains with a number of hydrogen bonds that can hold the molecule in position (Figure 6). Quercetin was able to dock to PKCδ in the same hydrophobic area (Figure 6A). Its binding modes to JAK2 kinase domains were almost identical, suggesting that quercetin may bind to and inhibit the function of both domains (Figure 6B). However, its binding mode was different to those of PKCδ.

Taken together, our findings provide evidence that quercetin attenuates UV-stimulated MMP-1 and COX-2 expressions via direct inhibition of JAK2 and PKCδ kinase activities, thereby preventing UV-induced skin aging.

## 3. Discussion

Quercetin, found in red wine, fruits, and vegetables, exhibits beneficial effects against various diseases such as diabetes, cancer, and inflammatory disorders [14,15]. However, little is known about its anti-skin aging effect and the target molecule of quercetin in UV-mediated signaling. Many studies have reported that UV-induced inflammatory responses and degradation of extracellular matrices are the primary mechanisms responsible for the development of skin aging [4,19]. We discovered that quercetin could block UV-induced COX-2, MMP-1, and collagen degradation in human skin. Examination of the molecular mechanism revealed that quercetin exerts protective effects against UV-mediated skin aging via directly targeting PKCδ and JAK2.

UV exposure induces MMP-1 expression, collagen degradation, and inflammation by activation of the transcription factors such as AP-1 and NF-κB [10,11,20]. AP-1 binds to specific regions of the promoter of *cox-2* and *mmp-1* [10]. Similarly, NF-κB is also known to regulate inflammatory related genes and *mmp-1* [11,21]. In our study, quercetin suppressed *cox-2* promoter activity, as well as AP-1 and NF-κB activation. The potent inhibitory effect towards AP-1 and NF-κB could be a key reason for the observed anti-skin aging phenotype driven by quercetin.

Because quercetin inhibited MAPK, Akt, and STAT3 signaling pathways, we hypothesized that the molecular targets of quercetin could be upstream kinases of MAPK, Akt, and STAT3. JAK2 kinase is a well-known upstream regulator of STAT3, and the JAK-STAT pathway is heavily involved in inflammation and growth regulation [12,13]. In addition, PKCδ is an upstream regulator of the MAPK and Akt signaling pathways [7,22]. To confirm the role of PKCδ in UV-induced MAPK signal pathway in skin cells, a PKCδ dominant negative protein was used in our study. Our results showed that PKCδ was involved in controlling UV-stimulated MAPK and Akt signaling.

PKCδ regulates various cellular functions including growth, proliferation, and inflammatory processes [23]. In addition, a previous study has shown that PKCδ modulates the expression of collagen genes in skin cells [24]. Novel PKC inhibitors have been shown to mediate UV-induced MMP-1 expression in a previous study [25]. JAK2 and PKCδ are involved in not only skin aging but also oncogenic alterations such as cell survival, proliferations, and invasion [23,26]. For these reasons, our results carefully suggest that the anti-cancer effect of quercetin may be partly related to the inhibition of JAK2 and PKCδ.

Our findings demonstrate the effect of quercetin on target molecules for UV-induced skin aging, highlighting its potential as a cosmeceutical ingredient for anti-aging products.

## 4. Materials and Methods

### 4.1. Reagents

Quercetin was purchased from Sigma-Aldrich (St. Louis, MO, USA). Antibodies for phospho-ERK, phospho-p38, phospho-JNK, phospho-Akt, phospho-STAT3, JNK, p38, ERK, Akt, STAT3, JAK2, and PKCδ were purchased from Cell Signaling Technology (Danvers, MA, USA). Quercetin (95% purity) was dissolved in DMSO (20 mM). Stock samples stored at −20 °C were used for the experiments for stability.

### 4.2. Cell Culture and UV Irradiation

The JB6 P+ and JB6 P+ PKCδ DN cells (kindly provided by Dr. Zigang Dong, University of Minnesota) were cultured in MEM (Corning, New York, NY, USA) containing 5% FBS (Gibco, Waltham, MA, USA) and 1% penicillin/streptomycin (Corning, New York, NY, USA) in a 37 °C incubator under a humidified atmosphere of 5% CO_2_. The solar UV light system (Q-Lab Corporation, Cleveland, OH) was used as the UV light source. Cells were exposed to UV at 26 KJcm^−2^ for 26 min. The percentage of UVA and UVB from lamps was measured with UV meter as 94.5% and 5%, respectively.

### 4.3. Ethic Statement

Human abdominal skin tissue was purchased from Biopredic International (Rennes, France) followed the French Law L.1245-2 CSP. The French Ministry of Higher Education and Research has approved Biopredic International, a human skin supplier, for the acquisition, transformation, sales, and export of human biological material to be used in research (AC-2013-1754, 13^th^ July 2018). This study complied all principles set forth in the Helsinki Declaration.

### 4.4. Excised Human Skin and UV Irradiation

Skin tissue (diameter of 10 mm) was incubated with DMEM (Corning, New York, NY, USA) containing 10% fetal bovine serum (Gibco, Waltham, MA, USA) with penicillin/streptomycin (Corning, New York, NY, USA) at 37 °C in a humidified incubator containing 5% CO_2_. Ex vivo human skin tissues were treated with quercetin and exposed to UV daily for 10 consecutive days.

### 4.5. COX-2 Promoter Reporter and AP-1 and NF-κB Activation Luciferase Assays

JB6 P+ cells stably transfected with AP-1, NF-κB, and COX-2 luciferase reporter were generated as previously described [27]. Luciferase activity was measured using the Steady-Glo® Luciferase Assay System (Promega, Madison, WI, USA) according to the manufacturer’s instructions. Luminescence was measured with a Varioskan Lux Multimode Microplate Reader (Thermo Fisher Scientific, Waltham, MA, USA).

### 4.6. Kinase Assays

The in vitro kinase assays were conducted using the SelectScreen Kinase profiling service (Thermo Fisher Scientific, Waltham, MA, USA).

### 4.7. Immunoblotting

After cell lysis, protein concentrations were quantified using a Pierce BCA Protein Assay Kit (Thermo Fisher Scientific, Waltham, MA, USA) as described in the manufacturer’s manual. Lysate proteins from cell and human skin tissues were separated by SDS-PAGE and transferred to a nitrocellulose membrane (Bio-Rad, Hercules, CA, USA). After blocking in 5% skim milk in TBS, containing 0.1% Tween 20 (TBST) the membrane was incubated with corresponding antibodies at 4 °C overnight. After washing with TBST, a horseradish peroxidase-conjugated secondary antibody was applied to the membranes and protein bands were visualized with Western Lightning Plus-ECL (Perkin Elmer, Waltham, MA, USA).

### 4.8. Histological Analaysis

Human skin tissues were fixed with 4% formaldehyde and embedded in paraffin. The sliced sections were stained with Masson’s trichrome for collagen fibers.

### 4.9. Preparation of Quercetin-Sepharose 4B Beads

Sepharose 4B powder (0.3 g) (GE Lifesciences, Piscataway, NJ, USA) was activated with 1 mM HCl and quercetin was conjugated to the activated Sepharose 4B beads in coupling solution (0.1 M NaHCO_3_, pH 8.3, and 0.5 M NaCl) by rotation overnight at 4 °C. The coupling solution was used to wash out the mixture, which was then transferred to 0.1 M Tris-HCl buffer (pH 8.3). The uncoupled-quercetin was washed out with washing buffer (0.1 M acetate buffer (pH 4.0) and 0.1 M Tris-HCl buffer (pH 8.0) containing 0.5 M NaCl).

### 4.10. In Vitro and Ex Vivo Pull-Down Assays

Lysates from JB6 P+ cells and ex vivo skin tissue samples were mixed with quercetin-conjugated Sepharose 4B beads or with Sepharose 4B beads alone (as a control) in reaction buffer (150 mM NaCl, 5 mM EDTA, 50 mM Tris pH 7.5, 1 mM DTT, 1 μg protease inhibitor mixture, 0.02 mM PMSF, 2 μg/mL bovine serum albumin and 0.01% Nonidet P-40) at 4 °C overnight with rotation. After washing the beads five times with washing buffer (150 mM NaCl, 5 mM EDTA, 50 mM Tris pH 7.5, 1 mM DTT, 0.02 mM PMSF, and 0.01% NP-40), binding was detected by immunoblotting with the corresponding antibodies.

### 4.11. Complex Structure Computational Modeling

A docking simulation was executed with AutoDock Vina (Version 1.1.2, The scripps Research Institute, La Jolla, CA, USA) [28] using three dimensional structures of quercetin, PKCδ, and JAK2. The structure of quercetin was retrieved from PubChem [29] (PubChem ID: 5280343), and the structures of the JAK2 kinase domains corresponding to residues 545~809 (PDB ID: 5UT3) and 849~1124 (PDB ID: 4D1S) were retrieved from the Protein Data Bank [30]. The structural model of PKCδ in the SWISS-MODEL repository [31], which was based on the structure of the homologue with a sequence identity of 72%, was used in the docking simulation. Up to 10 docking poses were sought within the cubic box of size 30 Angstroms placed near the ATP binding site, and the optimal docking pose was reported in this study.

### 4.12. Statistical Analysis

Data are expressed as mean ± standard deviation (SD) of three independent experiments. Statistical significance between groups was determined using ANOVA with post-hoc Bonferroni test using SPSS (Ver. 20; SPSS, Inc., Chicago, IL, USA). A value of *p* < 0.05 was considered to indicate statistical significance.

## 5. Conclusions

Our findings provide evidence that quercetin directly targets PKCδ and JAK2 protein kinases to effectively inhibit the hallmarks of UV-induced skin aging including wrinkle formation and inflammation in human skin tissues, and may have applications as a cosmeceutical ingredient to prevent skin photoaging.

## Supplementary Materials

Supplementary materials can be found at https://www.mdpi.com/1422-0067/20/21/5262/s1.

## Abbreviations

UV	Ultraviolet
MMP-1	Matrix metalloprotease-1
COX-2	Cyclooxygenase-2
NF-κB	Nuclear factor-κB
AP-1	Activator protein-1
MAPK	Mitogen-activated protein kinase
ERKs	Extracellular signal-regulated kinases
JNKs	c-Jun N terminal kinases
PKC-delta	Protein kinase C delta
JAK2	Janus kinase 2
STAT-3	Signal transducer the and activator of transcription 3
