# Supplementary material for: Quercetin Directly Targets JAK2 and PKCδ and Prevents UV-Induced Photoaging in Human Skin
Source: Int J Mol Sci. 2019 Oct 23;20(21):5262. doi: 10.3390/ijms20215262 (PMC6862686; doi:10.3390/ijms20215262)
Supplement: Supplementary file 1 [file ijms-20-05262-s001.pdf]

Supplementary figure

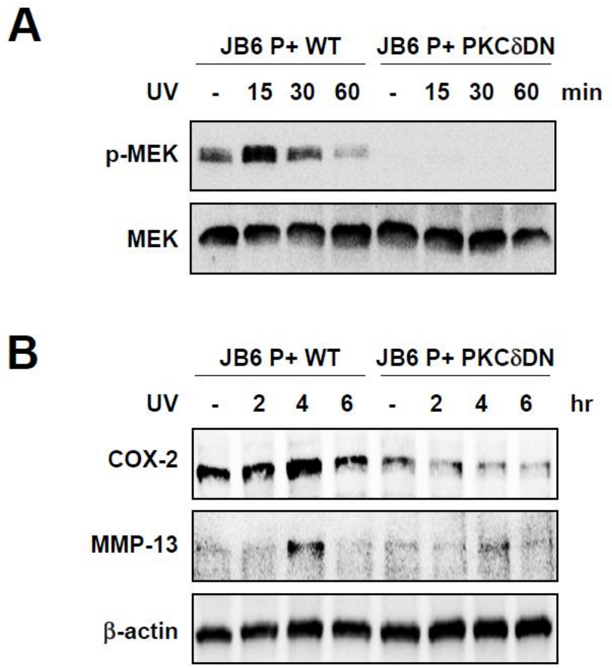

Figure S1. (**A**) Phosphorylation of MEK is reduced in PKCδDN cells. Phospho-MEK level was determined to confirm reduced activity of PKCδ (**B**) UV-induced COX-2 and MMP-13 expression levels are suppressed in PKCδDN cells. Mouse MMP-13 was used as a functional homologue of human MMP-1.
